# Effectiveness of Visual vs. Acoustic Closed-Loop Stimulation on EEG Power Density during NREM Sleep in Humans

**DOI:** 10.3390/clockssleep2020014

**Published:** 2020-04-30

**Authors:** Konstantin V. Danilenko, Evgenii Kobelev, Sergei V. Yarosh, Grigorii R. Khazankin, Ivan V. Brack, Polina V. Miroshnikova, Lyubomir I. Aftanas

**Affiliations:** 1 Institute of Physiology and Basic Medicine, 630117 Novosibirsk, Russia; kobelev13@mail.ru (E.K.); yarosh@physiol.ru (S.V.Y.); khazankin@physiol.ru (G.R.K.); brack@physiol.ru (I.V.B.); pvmirosh@gmail.com (P.V.M.); liaftanas@gmail.com (L.I.A.); 2 Department of Information Technologies, Novosibirsk State University, 630090 Novosibirsk, Russia; 3 V. Zelman Institute for Medicine and Psychology, Novosibirsk State University, 630090 Novosibirsk, Russia

**Keywords:** healthy subjects, NREM sleep, delta wave power density, acoustic stimulation, visual stimulation

## Abstract

The aim of the study was to investigate whether visual stimuli have the same potency to increase electroencephalography (EEG) delta wave power density during non-rapid eye movement (NREM) sleep as do auditory stimuli that may be practical in the treatment of some sleep disturbances. Nine healthy subjects underwent two polysomnography sessions—adaptation and experimental—with EEG electrodes positioned at Fz–Cz. Individually adjusted auditory (pink noise) and visual (light-emitting diode (LED) red light) paired 50-ms signals were automatically presented via headphones/eye mask during NREM sleep, shortly (0.75–0.90 s) after the EEG wave descended below a preset amplitude threshold (closed-loop in-phase stimulation). The alternately repeated 30-s epochs with stimuli of a given modality (light, sound, or light and sound simultaneously) were preceded and followed by 30-s epochs without stimulation. The number of artifact-free 1.5-min cycles taken in the analysis was such that the cycles with stimuli of different modalities were matched by number of stimuli presented. Acoustic stimuli caused an increase (*p* < 0.01) of EEG power density in the frequency band 0.5–3.0 Hz (slow waves); the values reverted to baseline at post-stimuli epochs. Light stimuli did not influence EEG slow wave power density (*p* > 0.01) and did not add to the acoustic stimuli effects. Thus, dim red light presented in a closed-loop in-phase fashion did not influence EEG power density during nocturnal sleep.

## 1. Introduction

Real-time modulation of sleep is an area of research targeted to modify/normalize architecture, duration, and/or depth of sleep by external stimuli, especially important in conditions characterized by sleep disturbances, such as psychophysiological insomnia and depression.

The two distinct states of non-rapid eye movement (NREM) and rapid eye movement (REM) sleep have their own neuroanatomic, electrophysiological, and behavioral characteristics. NREM is characterized by electroencephalography (EEG) that shows high-voltage K-complexes and sleep spindles in shallow sleep (stage N2) and high-voltage slow waves (0.5–2 Hz, amplitude above 75 µV) in deep, “slow-wave” sleep (stage N3) [1]. REM is characterized by low amplitude, mixed frequency EEG without K-complexes or sleep spindles [1]. NREM is followed by REM, with 4–5 of such cycles over the sleep episode, and the proportion of REM increasing towards the end of the night [2]. 

The voltage of the registered bioelectrical activity is normally highest in the first-cycle NREM sleep and then decreases exponentially [3]. A special parameter, EEG power density, calculated as square of the wave amplitude divided by wave frequency, namely in the frequency range between 0.5–4.5 Hz, is a recognized indicator of the effective dissipation of the ‘sleep pressure’ accumulated during daytime [3]. The dissipation is often attenuated in depression (reduced delta sleep ratio [4,5]), whereas increased NREM power density might be associated with better restorative sleep [2]. EEG power density is denoted as “slow wave activity” (SWA) in the frequency band 0.5–4 Hz (subdivided further on slow oscillation 0.5–1 Hz and delta waves 1–4 Hz) and “spindle activity” in the 9–15 Hz band (subdivided further on slow spindle 9–12 Hz and fast spindle 12–15 Hz) [6]. The increase in SWA is accompanied by a decrease in spindle band power density that is also known to be characteristic for (deeper) sleep [7].

Stimuli studied for their potency to modulate sleep in a real-time manner include acoustic, visual, or olfactory stimuli, electrical or magnetic transcranial stimulation, vestibular, somatosensory stimuli (of peripheral nerves) [8], or their combination (e.g., [9]). Repeated acoustic short (50 ms) stimuli increase slow wave activity and the number of K-complexes (reviewed in [10]). Modern techniques allow anchoring the short stimuli to the up phase of endogenous slow waves to increase slow oscillatory activity (closed-loop in-phase stimulation [11]), whereas stimulation time-locked to the down phase reduces slow waves [12]. The behavioral and physiological consequences of acoustic enhancement of sleep slow waves may include an improvement of verbal declarative memory [11,13,14,15] and immune status [16] in healthy adults.

Sleep modulation properties of stimuli other than acoustic have been less investigated in humans. Visual pulsatile stimulation was reported to be not as potent [8], though there are no published studies found. However, in one study a repeated light stimulation evoked more K-complexes than auditory stimuli did, probably due to the relatively high brightness of the stimuli presented from a light source positioned at three feet from the subject’s face [9]. Approximately 6% of light (5.6% of red, 0.3% of green, and 0.3% of blue light) passes through closed human eyelids [17], and even extremely low intensity light (0.002 lux) is sufficient to initiate a cascade of photochemical reactions in the open eyes of rats [18]. It is also known that blue light, in comparison to red light, may immediately increase energy/alertness levels in humans (when acting via the open eyes) [19,20].

The current investigation was done before the start of our next planned stimulatory study in depression. We aimed to investigate in healthy subjects of different ages whether visual stimuli have the same potency to increase delta wave power density during sleep as auditory stimuli do, and if the effects were synergistic, to implement both stimuli in depressed patients.

**Figure 1:**
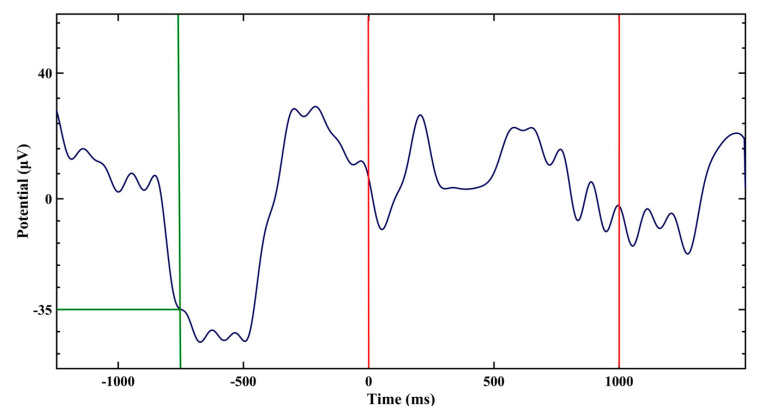
Schematic presentation of paired stimuli (red lines) programmed to start during stimulation epochs whenever the electroencephalography (EEG) wave crossed an established threshold (−35 µV for representative study participant #9, green lines). After the crossing, the first stimulus (red line at “0” time) in this subject was set to be delivered in 0.5 s (but occurred in 0.75 s in fact, see Section 2 for an explanation) to fall on an up-phase of the slow wave; the second stimulus was set to be delivered 1 s after the first.

The study comprised adaptation and experimental nights during which short (50 ms) paired (1–1.3 s apart; Figure 1) stimuli of a given modality (light, sound, or light and sound simultaneously) were automatically presented (in-phase with delta waves) within 30-s epochs preceded and followed by 30-s epochs without stimulation, and these 1.5-min blocks were repeated alternately (in the sequence ‘light’–’sound’–’light and sound’) throughout the night during NREM sleep.

## 2. Results

The study was performed in 2016–2017. Ten subjects completed the study, in nine of them (2 males, 7 females; mean age 32.3 years, range 21–46 years; Table 1) the obtained data on the experimental night were sufficient for the analysis. Time distribution of sleep stages was normal, with stages N2–3 comprising 74.8 ± 4.7% (mean ± SD) of sleep, and stage N1, REM, and wake/movement arousals altogether comprising 25.2 ± 4.7%. The majority of the stimuli fell on stages N2–3 (88.7 ± 3.4%). 

The amplitude threshold for the stimuli triggering (set after the adaptation night) varied inter-individually from −35 to −25 µV (Table 1), stimuli latency after the triggering from 0.5 to 0.65 ms, and stimuli intensity from 1 to 1.4 lx for light and from 43 to 46 dB for sound. The number of paired stimuli per EEG cycle ranged from 3 to 8. The number of cycles of each modality selected for the analysis ranged from 4 to 15 (stage N2, median = 9) and from 4 to 21 (stage N3, median = 11) (Table 1).

**Figure 2:**
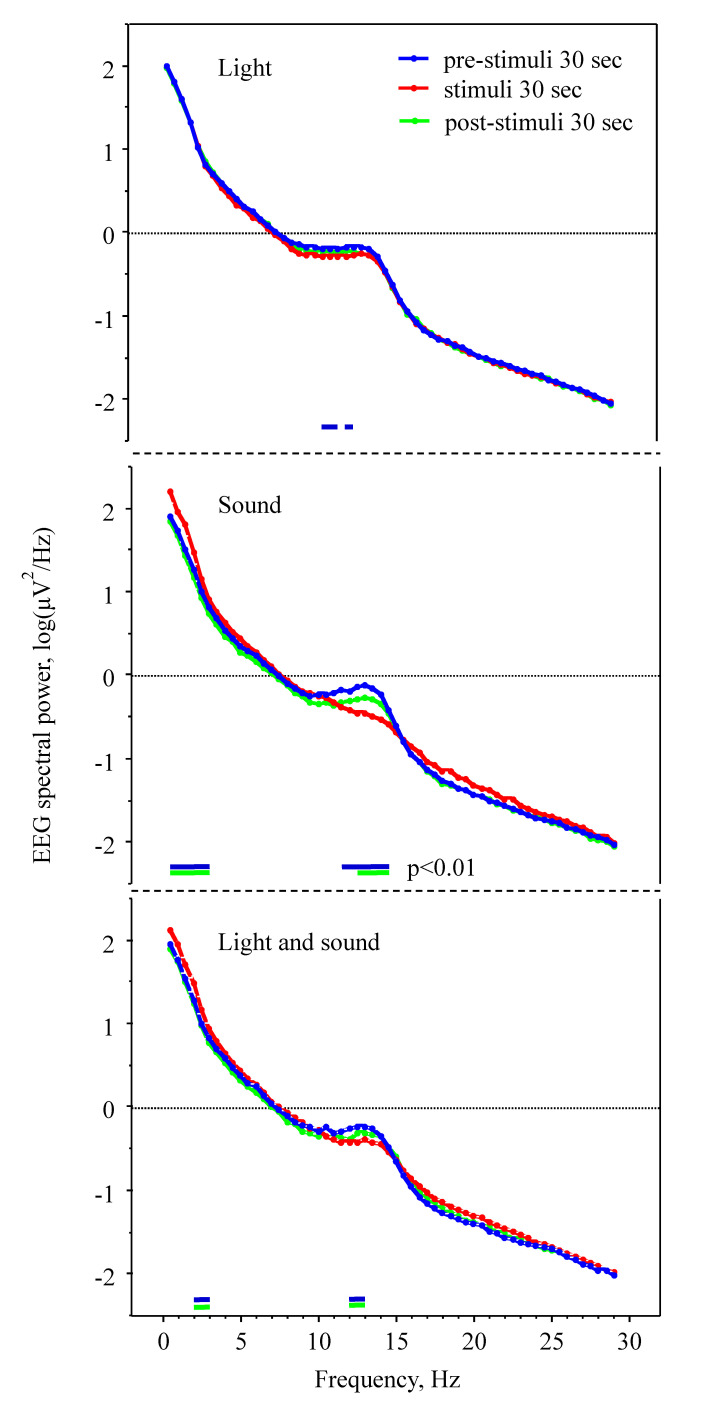
Effect of visual and/or acoustic stimulation on EEG spectral power density during sleep stage N3 in 9 healthy subjects. Significant differences (*p* < 0.01, Student’s *t*-test) are indicated by short lines beneath each diagram (blue line: stimuli vs. pre-stimuli epochs, green line: stimuli vs. post-stimuli epochs).

Light stimuli did not increase EEG slow wave (between 0.5–4 Hz) power density (*p* > 0.05 compared to the pre-exposure epochs, Student’s *t*-test) but slightly decreased power density at 10.5–13.5 Hz (*p* < 0.01; stage N3; Figure 2). Acoustic stimuli caused an increase (*p* < 0.01) of EEG power density in the frequency band 0.5–3.0 Hz (slow waves) and a decrease at 11.5–14.5 Hz (*p* < 0.01; fast spindle range); the values reverted to baseline at post-stimuli epochs. Simultaneous light and acoustic stimuli effects did not differ from the acoustic stimuli effects (*p* > 0.05). In neither condition did pre- and post-stimuli epoch values differ significantly (*p* > 0.01).

**Figure 3:**
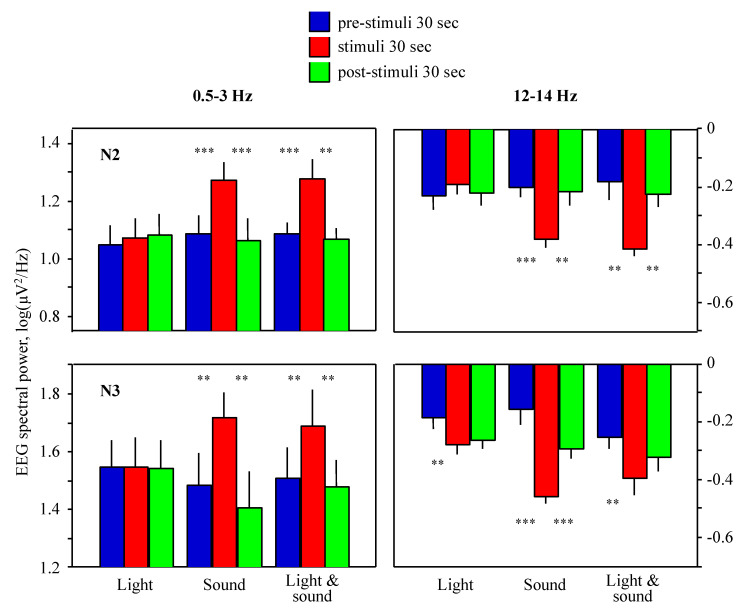
Effect of visual and/or acoustic stimulation (X-axis) on EEG power density (Y-axis) in frequency bands 0.5–3.0 Hz (left diagrams) and 12–14 Hz (right diagrams) during sleep stages N2 (upper diagrams) and N3 (lower diagrams). Bars indicate mean EEG power density over 0.5 Hz frequency bands and then over 9 healthy subjects, lines at the bars indicate standard errors of the means. EEG power density values at 30-s epochs with stimulation (red bars) were compared with pre-stimuli and post-stimuli epochs by Student’s *t*-test; ** *p* < 0.01, *** *p* < 0.001.

EEG power density values in the post-selected frequency bands 0.5–3.0 Hz and 12–14 Hz were pooled to be presented as bars with standard errors to firmly see the significant differences (Figure 3). Statistical analysis using repeated-measures ANOVA showed significant (*p* < 0.001) interactions of factors ‘Condition’ (light, sound, light and sound) and ‘Time’ (pre-stimuli, stimuli, post-stimuli) allowing post-hoc comparison with Student’s *t*-test. Sleep N2 stage analysis showed results similar to sleep N3 stage (Figure 3).

**Figure 4:**
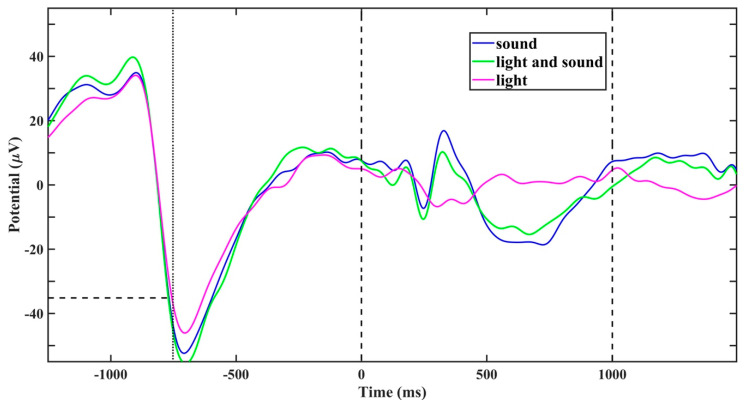
Average EEG waveforms, referenced to the time of the first (of two paired) stimuli presentation (vertical dashed line at 0 ms), time-locked to the detection of EEG-wave threshold (−35 µV for this representative subject #9; vertical line at −750 ms). The sound and light and sound stimuli evoked distinct event-related potential (ERP) that included all the classic ERP components (P200, N350, P450, N550, and P900 [11,21]).

Post-hoc analysis of the average EEG waveforms in response to the stimuli (event-related potential, ERP) revealed that, in spite of our intension, the stimuli latency after the triggering (set to 0.5–0.65 s) was constantly delayed for 0.25 s (due to computer system delay), and therefore, the stimulus did not fall on a slow wave peak but rather somewhat later, on the decay part of the slow wave up-phase (Figure 4). Even with such delay, our results demonstrate an increase in power density following stimulation.

## 3. Discussion

The study confirmed earlier findings that short acoustic repeated stimuli may increase EEG power density in slow wave NREM sleep [10]. This effect is accompanied by a decrease in fast spindle band power density that is known to be characteristic for (deeper) sleep [7]. In contrast to the acoustic stimuli of the same duration and frequency, dim red light presented in the same closed-loop in-phase fashion did not influence slow wave EEG power density, and there was no synergistic effect of these two stimuli on the EEG.

It is possible that the negative result may be related to too few, bipolar EEG electrodes being positioned over the frontal-parietal area, excluding the occipital lobe to which visual signals project. Although EEG slow waves are a global cortical phenomenon during sleep [22], slow waves may be different in amplitude and phase across different brain regions. Slow oscillations during NREM sleep propagate from the medial frontal cortex to the medial temporal lobe [23] and can be regulated locally [24,25]; therefore, the effect following the stimuli presentation may be attributable to a particular brain region only [26]. It is not excluded, therefore, that the slight EEG fluctuations around baseline following the light stimulus (Figure 4) may indeed represent visual-evoked potential (VEP) that would be more visible at the occipital region.

It is also possible that light will be effective if it acts via non-rod, non-cone, melanopsin photoreceptors in the retina, the projection of which are various deep brain structures [27]. These photoreceptors are sensitive to blue light at ~480-nm wavelength [27], while closed eyelids filter out blue and transmit mainly red light [17]. Theoretically, only bright light (containing blue wavelengths) may stimulate melanopsin through closed eyelids, and this might explain its efficacy in a study reported by Riedner et al. [9]. It appears that bright blue or green light may suppress melatonin secretion when applied via closed eyelids during sleep [28]. However, the use of bright light is impractical due to the awakening effect. Our pilot trial showed that green light-emitting diodes (LEDs) installed in the mask instead of red LEDs did not potentiate slow wave power density either. Nevertheless, a systematic study of the effects of light on sleep EEG depending on the EEG spatiality and light (spectral) characteristics is required, since the absence of the knowledge from such a basic study limits the strength of our conclusion about light inefficiency.

Following the acoustic stimulation, we received event-related responses containing spectral components (P200, N350, P450, N550, and P900) that are characteristic of stimulation-evoked K-complexes [21]. A convincing explanation of the closed-loop stimulation effects is that K-complexes externally evoked by stimulation time-locked to a particular phase of slow waves promote further synchronization of endogenous slow waves [8,26]. In our study, acoustic stimuli were delivered at the decay part of the slow wave up-phase in a relatively broad time range of the excitatory up-state of slow waves. The large range of time phase values at which the pulses are applied might be an advantage in the stimulation, considering that realistic neural oscillators do not have one determined natural resonant frequency, and a range of time-locked values (time delay) for these frequencies would also be effective.

## 4. Materials and Methods 

The study was approved by the local Ethics Committee (protocol #9 of 25.08.2016). The test subjects had to be between 20–50 years old and report good general health and normal sleep. The subjects were paid for their participation.

The study comprised two polysomnographic (PSG) night sessions—adaptation and experimental—with at least one off-protocol night in-between. Seven electrodes were positioned: 5 on the face (2 for oculography, 2 for electromyography, and 1 ground) and 2 on the scalp (for EEG), at Fz–Cz positions (midline frontal and midline central); bipolar derivations were used. Such a montage was due to the limited number of channel inputs (*N* = 3) in the compact polygraph Boslab BI-012 with the Boslab software (COMSIB LLC, Novosibirsk, Russia) used for the recording of the electrical biosignals. EEG electrode positions accounted for the fact that slow wave sleep (SWS) and the effect of stimulation on SWS has a frontal predominance [22]. The polygraph was programmed to communicate via API (Application Programming Interface) with a custom-made programmable stimulator AVSPlayer213 (Institute of Physiology and Basic Medicine, Novosibirsk, Russia) that triggered auditory and visual signals and could online set their intensity (0%–100%).

Auditory (pink noise) and visual (LED red light) paired 50-ms signals were automatically presented via in-ear headphones and thin cloth eye mask, respectively. The mask comprised 4 small LEDs for each eye between the mask layers and could provide light intensity up to 7.4 lux (luxmeter CEM DT-1309). Red light was chosen to avoid a possible effect of blue-green light to increase energy/alertness levels [19,20]. The stimuli onset was anchored to the moment when the program detected a decline of EEG waves below an amplitude threshold, with the elapsed time ~0.6 s to fall at the peak of the EEG slow wave (closed-loop in-phase stimulation as per [11]; Figure 1). The silent period after the onset (until the next paired stimuli triggering) was set to 2 s.

The EEG amplitude threshold, stimuli intensity, and stimuli latency were chosen individually based on the adaptation night recording. The amplitude threshold was adjusted (using Boslab software) during the adaptation night such that there were 3–8 paired stimuli over the 30-s epoch. The stimuli intensity was chosen from a variety of tested intensities (each presented within at least a 30-min period) as a maximal intensity that did not cause sleep movements or awakenings (verified during registration online, PSG analysis offline, and interview with the test subject after sleep). The stimuli latency was chosen as a half median of the determined (with a precision of 0.1 ms) peak frequency of slow waves 0.5–2 Hz (but had to be no less than 0.5 ms) and was set for the experimental night using the AVSPlayer213 software. The inter-stimulus interval was twice the latency time.

The 30-s epochs with stimuli of a given modality (light, sound, or light-and-sound simultaneously) were preceded and followed by 30-s epochs without stimulation, and these 1.5-min cycles, in turn, were presented alternately in the sequence ‘light’–’sound’–’light and sound’ cycles throughout the night. The stimuli would not be generally presented during REM sleep or stage N1 of NREM sleep as the EEG amplitude during these episodes was usually lower than 50 µV.

Sleep stages were scored in 30-s steps according to the American Academy of Sleep Medicine (AASM) classification rules [1]. EEG power density in these 30-s epochs was calculated for every 0.5-Hz band in the frequency range 0.5–30 Hz and log-transformed. Artifact-free cycles of sleep N3 stages were analyzed. The number of cycles taken in the analysis was such that the cycles with stimuli of different modalities were matched by number of stimuli presented. The same procedure was done for the sleep N2 stages.

The statistical analysis was performed using StatView 5.0.1 software and included paired Student’s *t*-test and analysis of variance for repeated measures (rANOVA). Differences between the EEG spectral power density values were considered to be statistically significant at probability level *p* < 0.01 (accounting for multiple comparisons and to reach a greater effect size when a small number of subjects is studied).

## 5. Conclusions

The study showed that the electroencephalography (EEG) slow wave power density during human NREM sleep (stages 2 and 3) was not influenced by dim red light (short 50-ms signals presented via the eye mask), whereas auditory stimuli presented in the same closed-loop in-phase fashion (via headphones), did increase the EEG slow wave power density, as previously reported. The study does not support practicality of the use of these visual stimuli in the treatment of sleep disturbances.

## Figures and Tables

**Table 1:** Characteristics of test participants and stimulation epochs.

Subject #	Gender	Age, y	Amplitude Threshold, µV	Sleep N2 Stage		Sleep N3 Stage	
				# of Epochs/# of Stimuli per Epoch ^1^	# of Epochs Taken in Analysis ^2^	# of Epochs/# of Stimuli per Epoch ^1^	# of Epochs Taken in Analysis ^2^
1	female	41	−25	38/4.1	6 × 3	34/5.7	4 × 3
2	male	33	−25	50/4.2	10 × 3	56/6.2	9 × 3
4	male	46	−25	59/4.3	12 × 3	44/6.3	7 × 3
5	female	43	−25	43/4.8	9 × 3	43/7.7	12 × 3
6	female	21	−25	35/5.1	5 × 3	79/7.4	21 × 3
7	female	40	−25	70/4.3	15 × 3	53/5.8	9 × 3
8	female	21	−30	31/4.5	4 × 3	62/6.6	11 × 3
9	female	22	−35	29/4.3	4 × 3	53/7.3	15 × 3
10	female	24	−30	53/4.7	12 × 3	44/7.5	11 × 3

^1^ Number of 30-s artifact-free sleep EEG epochs with stimulation (3–8 paired stimuli)/number of paired stimuli per epoch; ^2^ Number of epochs selected for analysis, equal across 3 stimulation modalities (light, sound, light and sound) and matched by number of stimuli presented per modality epoch.
